# Enhanced biofilm formation and multi‐host transmission evolve from divergent genetic backgrounds in *C* *ampylobacter jejuni*

**DOI:** 10.1111/1462-2920.13051

**Published:** 2015-10-14

**Authors:** Ben Pascoe, Guillaume Méric, Susan Murray, Koji Yahara, Leonardos Mageiros, Ryan Bowen, Nathan H. Jones, Rose E. Jeeves, Hilary M. Lappin‐Scott, Hiroshi Asakura, Samuel K. Sheppard

**Affiliations:** ^1^ College of MedicineInstitute of Life ScienceSwansea UniversitySwanseaUK; ^2^ MRC CLIMB ConsortiumInstitute of Life ScienceSwansea UniversitySwanseaUK; ^3^ Institute of Medical ScienceUniversity of TokyoTokyoJapan; ^4^ Division of Biomedical Food ResearchNational Institute of Health SciencesTokyoJapan; ^5^ Department of ZoologyUniversity of OxfordOxfordUK; ^6^ Present address: The Biostatistics CenterKurume University67 Asahi‐machiFukuoka830‐0011Japan; ^7^ Present address: Public Health England, Microbiology ServicesPorton DownSalisburyWiltshireSP4 0JGUK

## Abstract

Multicellular biofilms are an ancient bacterial adaptation that offers a protective environment for survival in hostile habitats. In microaerophilic organisms such as *C* *ampylobacter*, biofilms play a key role in transmission to humans as the bacteria are exposed to atmospheric oxygen concentrations when leaving the reservoir host gut. Genetic determinants of biofilm formation differ between species, but little is known about how strains of the same species achieve the biofilm phenotype with different genetic backgrounds. Our approach combines genome‐wide association studies with traditional microbiology techniques to investigate the genetic basis of biofilm formation in 102 *C* *ampylobacter jejuni* isolates. We quantified biofilm formation among the isolates and identified hotspots of genetic variation in homologous sequences that correspond to variation in biofilm phenotypes. Thirteen genes demonstrated a statistically robust association including those involved in adhesion, motility, glycosylation, capsule production and oxidative stress. The genes associated with biofilm formation were different in the host generalist ST‐21 and ST‐45 clonal complexes, which are frequently isolated from multiple host species and clinical samples. This suggests the evolution of enhanced biofilm from different genetic backgrounds and a possible role in colonization of multiple hosts and transmission to humans.

## Introduction

The formation of multicellular biofilms is an ancient adaptation shared by numerous bacteria and archaea (Otto, 2014; Solano *et al*., 2014). These structurally complex, dynamic systems offer a protective environment for survival in hostile habitats and can play a key role in the dispersal of pathogens. Biofilm formation involves the interaction of genetic and environmental factors and the relative contribution of these is the subject of ongoing debate. Much of the work in this area has focused on identifying biofilm‐associated genes such as those involved in surface adhesion, motility and regulation or expression of extracellular polymeric substances. Experiments typically involve comparison of wild‐type and mutant strains to assess the role of specific genes (Asakura *et al*., 2007; Svensson *et al*., 2009; Gundogdu *et al*., 2011; Frirdich *et al*., 2012; Sulaeman *et al*., 2012; Oh and Jeon, 2014), but understanding the influence of genetic variation in complex natural populations with multiple strains has been challenging. In particular, it has been difficult to quantify multi‐gene associations and the role of homologous sequence variation.

Recent advances in high‐throughput sequencing technologies and the increasing availability of genome‐sequenced isolate collections provide opportunities for investigating the genetic basis of complex traits. Genome‐wide association studies, which have been widely used in human genetics, can identify statistical associations between causal genetic variation and phenotype (Wellcome Trust Case Control Consortium, 2007; Craddock *et al*., 2010). These techniques have considerable potential for enhancing understanding of how genetic variation in natural bacterial populations may influence their ecology. However, there are specific challenges when applying genome‐wide association studies to bacteria (Sheppard *et al*., 2013a; Alam *et al*., 2014; Laabei *et al*., 2014). First, bacterial species can display a high degree of genetic variation within shared core genome elements and can have a large accessory genome. Second, because of the clonal population structure, the progeny of an expanding lineage will share adaptive elements associated with the phenotype of interest, as well as elements that are not, confounding the association and reducing the statistical power (Falush and Bowden, 2006).

Through simultaneous identification of core and accessory genome associations, and weighting with reference to the clonal frame of the population (Sheppard *et al*., 2013a), we identify genetic elements that are associated with biofilm production in the microaerophilic bacteria *Campylobacter jejuni* and *C. coli.* These organisms are common in the gut of several wild and agricultural animals (Sheppard *et al*., 2011), frequently infecting humans via contaminated meat and poultry, and are among the leading causes of food‐borne gastroenteritis worldwide (van Asselt *et al*., 2008). Passage through the food chain may be promoted by attachment to non‐biological surfaces and other cells, which is common in these species (Pearson *et al*., 1993; Trachoo and Frank, 2002; Zimmer *et al*., 2003; Bull *et al*., 2006), and can double the survival period outside of the host under atmospheric conditions (Joshua *et al*., 2006; Asakura *et al*., 2007).

*Campylobacter* populations are highly structured into clusters of related lineages, including deep branching clades (*C. coli*) (Sheppard *et al*., 2008; 2010) and clonal complexes of isolates that share four or more alleles at seven multilocus sequence typing (MLST) loci (Dingle *et al*., 2001). Characterization of thousands of isolates deposited in pubMLST (http://pubmlst.org/campylobacter/) identified clonal complexes that are typically only sampled from the gut of a single host species and can be termed host specialists. Other sequence types (STs), such as ST‐21 and ST‐45 clonal complexes, are found in multiple host species and can be termed host generalists (Sheppard *et al*., 2011). Although the significance of this has not – until now – been related to biofilm formation, there is evidence that *C. jejuni* strains differ in their capacity to form biofilms (Revez *et al*., 2011; Asakura *et al*., 2012). This could promote survival outside of the host, transmission and the colonization of multiple host species by important disease‐causing lineages (Sheppard *et al*., 2009).

## Results

### Differential biofilm formation in *C* *. jejuni* and *C* *. coli* isolates

**Figure 1:**
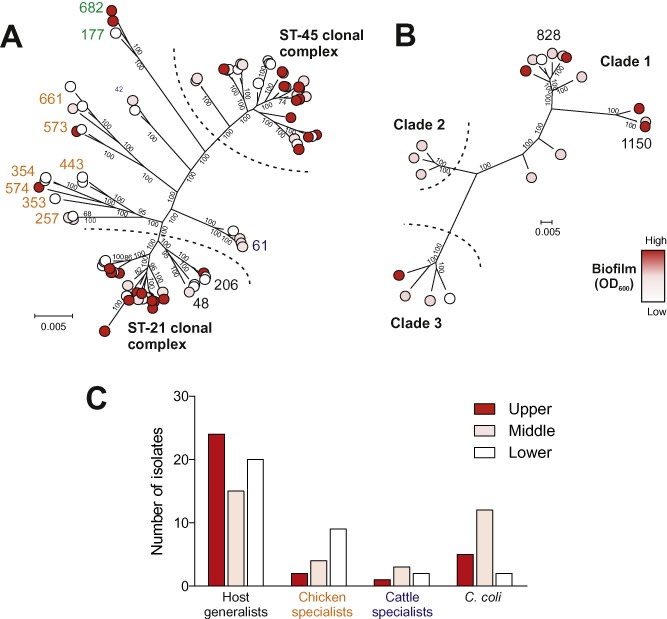
Genetic relatedness and biofilm formation of *C* *. jejuni* and *C* *. coli* isolates. Population structure of (A) *C* *. jejuni* and (B) *C* *. coli* with each isolate labelled as belonging to the upper (red; OD_600_ above 0.272), middle (pink; OD_600_ between 0.201 and 0.272) or lowest (white; OD_600_ below 0.201) 33rd percentile of biofilm formers based upon phenotype assays. MLST clonal complexes coloured according to the common isolate source for *C* *. jejuni* generalists (black), cattle specialists (blue), chicken specialists (orange) and wild bird specialists (green). Trees were based on 761 381 and 1 018 234 bp core genome alignments for *C* *. jejuni* and *C* *. coli*, respectively, calculated using an approximation of the maximum likelihood algorithm. The scale bar represents the average number of polymorphisms per site. Local support values on each branch were calculated with default values in FastTree2. Values reflect the reliability of each split in the tree and were estimated using the Shimodaira–Hasegawa test on the three alternate topologies around that split, at a default re‐sampling rate of 1000 re‐samples for each split. (C) Biofilm formation scores in four major ecological groups of *C* *ampylobacter* including host generalists, chicken specialists, cattle specialists and *C* *. coli*. The proportion of isolates in the upper, middle and lowest biofilm formation percentile in these four ecological groups is statistically different (χ^2^ test; *P* = 0.0116).

The extent of biofilm formation was measured from the absorbance value (OD_600_) of adhered cells following fixation and staining with crystal violet (Mack *et al*., 1994). Absorbance values (OD_600_) ranged from 0.065 to 1.005, with a mean reading for biofilm thickness of 0.263 and the variance of 0.022 (Fig. S1). Biofilm production was mapped onto the core genome trees for *C. jejuni* (Fig. 1A) and *C. coli* (Fig. 1B). Biofilm formation was not equally distributed across *Campylobacter* lineages (χ^2^ = 16.43, 6 degrees of freedom, *P* = 0.0116). A higher proportion (71%, 24 of 34) of the isolates that demonstrated biofilm production above an OD_600_ of 0.272 were host generalist *C. jejuni* isolates compared with cattle and chicken specialists. Roughly two‐thirds (64%, 9 of 14) of the chicken specialist isolates produced a biofilm reading of less than 0.201. Wild bird specialists were not found among the lower biofilm producers, although three isolates are not enough to draw any definitive conclusions (green labels on Fig. 1A). *C. coli* also showed an imbalance, with more Clade 1 isolates producing higher levels of biofilm (Fig. 1C).

Based on the frequency that MLST clonal complexes are isolated from different sources (http://pubmlst.org/campylobacter/) and published literature (Sheppard *et al*., 2014), *C. jejuni* isolates in this study (Table S1) were classified as chicken specialist isolates belonging to ST‐257 (*n* = 3), ST‐283 (*n* = 1), ST‐353 (*n* = 1), ST‐354 (*n* = 2), ST‐443 (*n* = 2), ST‐573 (*n* = 3), ST‐574 (*n* = 1) and ST‐661 (*n* = 3). Cattle specialists belonging to the ST‐61 (*n* = 4) and ST‐42 (*n* = 2) complexes and host generalists belonging to the ST‐21 (*n* = 26) and the ST‐45–206‐48 complexes (*n* = 33). *C. coli* isolates were classified according to three deep branching clades (Sheppard *et al*., 2010), with 12 isolates from Clade 1 and three isolates from Clades 2 and 3. In total, 50 unique STs from 19 clonal complexes were represented (Sheppard *et al*., 2011).

### Genetic elements associated with biofilm formation

**Figure 2:**
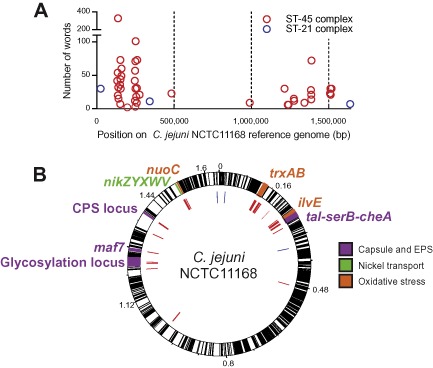
Genome position of biofilm‐associated genetic elements. A. Frequency of biofilm‐associated 30 bp words from the original and/or verification genome‐wide association studies, mapped to the *C* *. jejuni* NCTC11168 reference genome for ST‐21 (blue) and ST‐45 (red) clonal complex isolates. B. Visualized using Artemis (Carver *et al*., 2009) showing biofilm‐associated words (inner circle) in relation to annotated coding regions (black lines) and oxidative stress (orange), capsule and extrapolysaccharides (purple), nickel transport (green) and biofilm genes from published studies (Svensson *et al*., 2009; Howlett *et al*., 2012; Avila‐Ramirez *et al*., 2013; van Alphen *et al*., 2014).

The genetic association of enhanced biofilm formation was investigated in the two lineages containing isolates demonstrating a range in biofilm phenotypes, *C. jejuni* ST‐21 and ST‐45 clonal complexes (Fig. 1). A ClonalFrame tree of ST‐21 and ST‐45 clonal complexes were separately reconstructed using core gene‐by‐gene alignments for 23 of the highest biofilm producers (12 ST‐21 and 11 ST‐45 clonal complex isolates) and 18 of the lowest biofilm producers (7 ST‐21 and 11 ST‐45 clonal complex isolates). The genome‐wide association study was performed separately for the ST‐21 and ST‐45 clonal complexes, which identified 1657 30 bp words associated with enhanced biofilm formation. Words were mapped back to the reference *C. jejuni* NCTC11168 genome using BLAST to reveal areas and genes associated with biofilm formation (Fig. 2). There were 46 biofilm‐associated genes in total (Table S2).

The association gave different results when conducted using genomes from ST‐21 (*n* = 19) or ST‐45 (*n* = 22) clonal complex isolates. In ST‐21 clonal complex, three genes were found to be associated with enhanced biofilm production, while 43 genes were identified in ST‐45 clonal complex (Fig. 2, Table S2). The null distributions of the extent of association between presence of a word and enhanced biofilm formation given the clonal tree are shown in Fig. S2 for ST‐21 and ST‐45 clonal complexes, separately. The bimodal null distribution in ST‐21 indicates stronger population structure than that in ST‐45 clonal complex. This affects genome‐wide association (GWAS) power resulting in fewer hits for this clonal complex. However, significant hits satisfying *P* < 0.001 were still obtained in ST‐21.

A total of 605 words mapped to genes that have previously been shown to have a functional association with increased biofilm production, such as motility (Svensson *et al*., 2014), chemotaxis (Golz *et al*., 2012), capsule production (Malde *et al*., 2014) and protein glycosylation (Joshua *et al*., 2006; Guerry, 2007) (Table S2; highlighted in Fig. 2B). Additionally, 1052 associated words were mapped to other genes with a potential influence on biofilm formation in *Campylobacter.* These included genes putatively involved in biotin biosynthesis, cell wall biosynthesis, nickel transport, genes involved in heat shock, and iron or zinc uptake. At least four genes thought to be involved in sensing oxidative stress were also associated with biofilm production, including *trxA*, *trxB*, *ilvE* and *nuoC*. Some words could not be mapped back to the reference genome, but through comparison with the uncharacterized genomes from the original dataset we were able to map the 224 words to 43 putative genes. These genes displayed homology to known cell wall, capsule, oligosaccharide production, iron homeostasis and oxidative stress proteins.

A smaller collection of sequenced *Campylobacter* isolates were tested for biofilm formation and used as verification datasets. A second GWAS was run on both clonal complex isolates comparing the highest biofilm producers with the lowest (ST‐45 *n* = 14; ST‐21 *n* = 13; Table S1). Of the 46 genes that were previously found to be associated with biofilm formation, 13 were also identified using this verification dataset (*P*‐value, 0.01). These genes included several from the capsular polysaccharide (CJ1413c‐CJ1448c) and glycosylation loci (CJ1293‐CJ1342), which have a known effect on *Campylobacter* biofilm formation (Fig. 2, Table S2) (Guerry *et al*., 2006; Joshua *et al*., 2006).

### Exposure to atmospheric oxygen conditions enhances biofilm formation in *C* *. jejuni*

**Figure 3:**
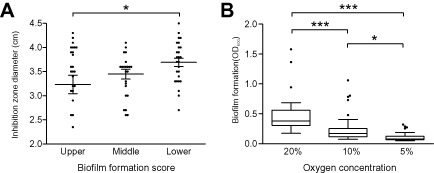
Correlation of biofilm formation with oxidative stress resistance phenotypes. A. Distribution of H_2_ O_2_ inhibition zone diameters (cm) on agar plates for isolates in the upper (above OD_600_ of 0.272), middle and lowest (below OD_600_ of 0.201) 33rd percentile of biofilm formation. Each point represents at least three biological replicates per isolate. The mean is indicated, with error bars representing the standard deviation. B. Distribution of biofilm formation (OD at 600 nm) at different oxygen concentrations (20% – atmospheric, 10% and 5%). Tukey box plots are shown, the horizontal bar represents the median and the box encompasses half of all data points for the corresponding condition. Asterisks in both panels indicate statistical significant differences between conditions with one asterisk for *P* ≤ 0.05, two for *P* ≤ 0.01 and three for *P* ≤ 0.001.

*Campylobacter* is a microaerophilic organism and resistance to oxygen stress in the extra‐host environment, by mechanisms other than biofilm formation, is likely to be advantageous for survival and transmission between hosts. Consistent with this, the association study based upon the biofilm phenotype identified four genes (with a *P*‐value below 0.001) with roles in protection from oxidative stress, which was tested with phenotyping assays for resistance to hydrogen peroxide (H_2_O_2_) and exposure to atmospheric oxygen (Fig. 3). As biofilm production abilities increased, the bacterial inhibition caused by disc diffusion assays with 3% H_2_O_2_ decreased, or the resistance to H_2_O_2_ increased (one‐way analysis of variance, *P* = 0.0652). This difference was statistically significant between isolates that were among the highest biofilm producers (above OD_600_ = 0.272) and lowest biofilm producers (below OD_600_ = 0.201; unpaired *t*‐test; *P* = 0.036; Fig. 3A).

Additionally, we compared the effect of exposure to oxygen under atmospheric conditions and with controlled atmospheric conditions (10% and 5% O_2_) on *in vitro* biofilm production (Fig. 3B). After correcting the measured values with the level of growth reached by all isolates under 20%, 10% and 5% O_2_ atmospheric concentration (Fig. S3), we observed that biofilm production on average was high (OD_600_ = 0.526) in natural atmospheric conditions, and steadily decreased (OD_600_ = 0.234 and 0.098) in the presence of 10% and 5% O_2_ (Fig. 3B). The increase of atmospheric concentration of O_2_ was accompanied by a proportional increase in biofilm production (Fig. 3B).

## Discussion

### Genome‐wide association study for complex resistance and survival traits

Biofilm formation is influenced by environmental factors, including multiple species interactions (Lee *et al*., 2014), as well as transcriptional variation (Wu *et al*., 2014) and phase variation (Brooks and Jefferson, 2014). However, differences in the genes associated with biofilm formation within species are less well characterized. Using an association study based on evolutionary model of bacterial evolution (Sheppard *et al*., 2013b), and traditional microbiology techniques, we provide insight into the genetic basis biofilm formation. The association study identified 46 genes associated with this complex trait, including genes involved in adhesion (Sulaeman *et al*., 2012), motility (Hendrixson *et al*., 2001; Kalmokoff *et al*., 2006), capsular polysaccharide production (van Alphen *et al*., 2014), nickel transport (Howlett *et al*., 2012; Avila‐Ramirez *et al*., 2013) and oxidative stress (Svensson *et al*., 2009), which have all previously been linked with biofilm formation (Table S2).

It is expected that false positives are also included in the results by multiple testing of the 30 bp words. However, the present study shows that high association scores are not randomly distributed around the genome, as would be expected by the null model, but instead enriched in certain genes. For example, about 37% of the words with *P* < 0.001 were found in genes that have previously been shown to have a functional association with increased biofilm production, which is less than 8% of all biofilm‐associated genes. The remaining words were also mapped to genes with a potential influence on biofilm formation.

Taken together, these genes were clustered on the genome, and included nine multi‐gene transcriptional units that have previously been described (Taboada *et al*., 2012), as well as other regions of associated elements. The colocalization of associated genes is influenced by genetic linkage. This is enhanced in recombining organisms, such as *Campylobacter*, because genetic exchange of biofilm‐associated elements can also affect adjacent sequence. Selection may also influence the evolution of gene order leading to coinheritance of elements of that confer adaptation to related environmental pressures. Consistent with this, genes putatively involved in sensing or responding to oxidative stress were over‐represented in biofilm‐forming isolates, as well as those directly related to biofilm formation. This link has previously been demonstrated with an *ahpC* mutant strain showing increased biofilm formation in response to oxidative stress (Oh and Jeon, 2014) and a mutant of the oxidative stress transcription regulator gene, Cj1556, showing reduced biofilm (Gundogdu *et al*., 2011). It is possible to identify candidate genes associated with complex traits, such as biofilm formation but further experiments are needed to directly link genes to function, particularly when growth conditions, such as the oxygen concentration can influence biofilm formation in *Campylobacter* (Sulaeman *et al*., 2012).

### Oxygen tolerance enhances transmission in host generalist *Campylobacter*

Our results show a higher proportion of isolates that were high biofilm formers (above OD_600_ = 0.272) among the two major host generalist clusters, which may suggest an association between oxygen tolerance in *Campylobacter* and the ability to colonize multiple hosts. Some high biofilm‐producing isolates are also present among lineages associated with chicken or wild bird hosts (Fig. 1). This implies that biofilm formation may also be important in host specialist lineages, or that biofilm formation has played a role in their recent ancestry. Furthermore, adhesion and clustering of bacteria can also influence survival within the host, for example, through interactions with the immune response that may favour certain lineages. Biofilms may also confer advantages in different contexts, but the role in survival outside of the host is known to be important in transmission and the promotion of host generalism in multi‐host bacteria (Woolhouse *et al*., 2001; 2005). Over time, this will lead to genomic differences that can be detected, for example among the isolates in this study.

### Host generalist *C* *ampylobacter* use different genes for enhanced biofilm formation

Some genes are associated with biofilm formation in multiple bacterial species. For example, polysaccharide production is known to be linked to biofilms in *Staphylococcus*, *Pseudomonas* and *Campylobacter* (Joshua *et al*., 2006; Rohde *et al*., 2010; Ghafoor *et al*., 2011; Spiliopoulou *et al*., 2012). Links with oxidative stress have been observed with biofilm formation in *Campylobacter* (Fields and Thompson, 2008; Oh and Jeon, 2014), *Yersinia* (Bobrov *et al*., 2007), *Staphylococcus* (Liu *et al*., 2013) and *Helicobacter* (Barnard *et al*., 2004). However, this is not always the case and, as with some other complex multigene functions, convergent phenotypes can be achieved through divergent genetic changes. Specific extracellular polysaccharide proteins such as Bap (Valle *et al*., 2012), Embp (Christner *et al*., 2010) and SasG in *S. aureus* (Rohde *et al*., 2007) have been associated with biofilm formation to varying degrees in different *Staphylococcal* species. *In vitro* studies of short‐term diversification and selection for mutations associated with biofilm production have shown extensive parallel evolution within strains of the same species, often involving identical nucleotides (McElroy *et al*., 2014). However, the role of variation in lineage‐specific fixed substitutions and accessory genome elements within species is largely uncharacterized.

In *Campylobacter*, biofilm formation was common to the two host generalist lineages but there were differences in the number and predicted function of the associated genes and alleles. Associated words from both GWAS were found in all other investigated clonal complexes (Fig. S4), but there was no overlap in the 46 biofilm‐associated genes from both GWAS. Three genes were associated with increased biofilm formation in ST‐21 complex isolates and 43 in ST‐45 complex isolates. A second GWAS was conducted on smaller verification datasets, which also identified 13 of the 43 biofilm‐associated genes in ST‐45 clonal complex isolates. None of the three biofilm‐associated genes were identified in the ST‐21 verification dataset. In spite of this, biofilm formation and oxidative stress phenotypes were similar. The difference in biofilm genes in these lineages with convergent phenotypes implies a complex genetic background to biofilm formation, possibly reflecting its fundamental importance. Host generalists can be classified as such based on their prevalence in different agricultural hosts and in humans, but the mechanisms involved in their host transmission pathways are poorly understood. Putative differences in the ecology of isolates can be implied from the different accessory gene content and the genetic isolation – despite being found in overlapping niches where recombination between some lineages is common (Sheppard *et al*., 2014).

## Conclusion

We present an integrated statistical genomics approach to understanding the genetic determinants of complex traits in bacteria. By comparing the genomes of 102 isolates and investigating phenotypic variation, we demonstrate that biofilm production increases protection from oxidative stress in host generalist *Campylobacter* more than in host specialist lineages, consistent with a role in host transmission ecology. The genes involved in biofilm formation differed between the two main host generalist *C. jejuni* lineages while the phenotypes remained the same. This raises questions about the evolution of biofilm formation, suggesting not only the involvement of numerous genes but also that different groups of genes can confer convergent phenotypes in isolates from the same species.

## Experimental procedures

### Bacterial isolates

An isolate collection was assembled, from the Swansea University archive, including 102 *C. jejuni* and *C. coli* isolates from various host species and clinical samples, including all isolates sequenced in Sheppard *et al*. (2013a, 2013b) (Table S1). This collection contained strains representing much of the genetic diversity among agricultural *C. jejuni* and the three major *C. coli* clades (Parkhill *et al*., 2000; Fouts *et al*., 2005; Pearson *et al*., 2007; Sheppard *et al*., 2013a, 2013b). These were supplemented with *Campylobacter* whole genome sequences from the NCBI database for genomic analyses (Genbank accession numbers: NC_009839; NC_008787; NC_003912; NC_002163) (Table S1). The isolate genomes are archived in the Dryad data repository (doi.org/10.5061/dryad.215jd/1 and doi:10.5061/dryad.28n35). Isolates were stored in a 20% (v/v) glycerol medium mix at minus 80°C and subcultured onto *Campylobacter* selective blood‐free agar (mCCDA, CM0739, Oxoid). Plates were incubated at 42°C for 48 h under microaerobic conditions (5% CO_2_, 5% O_2_) generated using a CamyGen (CN0025, Oxoid) sachet in a sealed container. Subsequent phenotype assays were performed on Brucella agar (CM0271, Oxoid).

### Genome archiving and core genome definition

Isolate genomes were archived in a web‐accessible database that supports functionality for identifying the gene presence and allelic variation by comparison to a reference locus list (Jolley and Maiden, 2010; Sheppard *et al*., 2012; Meric *et al*., 2014). This list comprised 1623 locus designations from the annotated genome of *C. jejuni* strain NCTC11168 (Genbank accession number: NC_002163.1) (Cabello *et al*., 1997; Parkhill *et al*., 2001; Gundogdu *et al*., 2007). These reference loci were identified in each of the 102 study genomes using BLAST. Loci were recorded as present if the sequence had ≥70% nucleotide identity over ≥50% of the gene length. The number of genes shared by all the isolates was defined as was a core genome for *C. jejuni* and *C. coli* separately.

### Phylogenetic trees and genealogy

From the core genome lists, 971 genes were shared by all *C. jejuni* (*n* = 83) and 1056 by all *C. coli* (*n* = 19). These genes were aligned individually for the 102 genomes, using MUSCLE (Edgar, 2004), and concatenated into a single multi‐FASTA alignment file for each isolate. Species core genome maximum likelihood trees were produced using FastTree2 with the generalized time reversible substitution model (Price *et al*., 2010), which allows the reconstruction of branch lengths greater than 0.0000005, corresponding to a minimum branch length of one substitution for every 2 000 000 base pairs (1000 times higher than the default FastTree parameters). The *C. jejuni* tree was created using 53 040 variable sites for a total alignment length of 761 381 bp. The *C. coli* tree was created using 141 461 variable sites for a total alignment length of 1 018 234 bp.

Isolates producing biofilm thickness in the upper 33rd percentile (OD_600_ ranging from 0.272 to 1.005, *n* = 34) and lower 33rd percentile (OD_600_ ranging from 0.065 to 0.201, *n* = 34) were compared using ClonalFrame, a model‐based approach to determining microevolution in bacteria, that accounts for the effect of homologous recombination (Didelot and Falush, 2007). The algorithm was run on 19 ST‐21 and 22 ST‐45 clonal complex isolate genome alignments, with 10 000 burn‐in iterations followed by 10 000 sampling iterations. These isolates were used in the GWAS.

### Phenotype testing

*C. jejuni* and *C. coli* are microaerophilic organisms and survival outside of the host gut is likely to involve interaction with other isolates and lineage‐specific variations. To address this, we quantified aspects of biofilm formation and tolerance to increasing oxygen levels. Attachment and accumulation of *Campylobacter* isolates was measured using a semi‐quantitative adherence assay using 96‐well tissue culture plates (Mack *et al*., 1994; Christner *et al*., 2012; Coffey and Anderson, 2014). Briefly, 200 μl of liquid media was inoculated with 5 μl aliquots of overnight culture (OD_600_ between 1.0 and 1.5) in a 96‐well plate. Plates were incubated on a moving platform at 42°C for 48 h in a sealed container under microaerobic conditions (∼5% O_2_, ∼ 10% CO_2_), maintained with a CampyGen atmosphere generation system (CN0025, Oxoid). Culture medium was removed and the wells washed with PBS. Adhered bacteria were fixed with 150 μl of Bouin's solution (7.5 ml picric acid; 2.5 ml 40% formaldehyde; 0.5 ml acetic acid) for 15 min and washed again with PBS. Plates were air‐dried and then stained with 150 μl of 0.1% (w/v) crystal violet for 5 min. Excess stain was removed, adhered bacteria were air‐dried and spectrophotometric measurements were taken at OD_600_ in a 96‐well plate reader and the average of at least three replicates was calculated (BMG Omega).

In addition, the biofilm phenotype was monitored in response to increasing oxygen concentrations for individual isolates in liquid culture (*n* = 41). An aliquot of 5 μl of overnight growth (OD_600_ between 1.0 and 1.5) in liquid medium from 2‐day‐old cultures on nutrient‐rich Brucella agar plates was used to inoculate 200 μl of fresh medium in a 96‐well plate. Cultures were grown and monitored under various controlled atmospheric conditions at 42°C in a BMG Omega plate reader with atmospheric control unit for 72 h. Tolerance to oxidative stress was tested on a subset of isolates (*n* = 77) by disc diffusion assay with hydrogen peroxide. Liquid cultures were streaked onto Brucella agar for confluent growth and filter discs containing a 3% (w/w) H_2_O_2_ solution were placed in the middle of the plate and incubated overnight at 42°C. Zones of inhibition were measured with a ruler (mm) and the mean zone of inhibition of three separate assays was noted. Average zones of inhibition ranged from 0 to 46 mm, with an overall mean zone of inhibition of 35 mm and a variance of 5 mm.

### Genome‐wide association mapping

Genetic elements associated with biofilm formation were identified using a genome‐wide association study approach (Sheppard *et al*., 2013a). Isolates producing biofilm thickness in the upper 33rd percentile (OD_600_ ranging from 0.272 to 1.005, *n* = 34) and lower 33rd percentile (OD_600_ ranging from 0.065 to 0.201, *n* = 34) were compared (Fig. S1). Biofilm formation was not a function of growth rate as isolates from all groups reached similar cell densities in liquid media after 72 h (Fig. S5).

The whole genome sequence of each isolate was fragmented into unique overlapping 30 bp words. This approach is alignment free and allows the simultaneous detection of genomic variation resulting from point mutation, homologous recombination and lateral gene transfer. For each word, we examined the extent of association based on a 2 × 2 table (with four cells *a*, *b*, *c*, *d*) in which rows indicate presence/absence of the word and columns indicate upper or lower biofilm formation, and calculated an association score as *a* + *d* – (*b* + *c*). To test significance of association of each word after controlling for the effect of population structure and clonal inheritance of genetic variants, the method computed *P*‐values by comparing the observed association score with a null distribution of the score (Fig. S2). The null distribution was created by a Monte Carlo simulation with 10^6^ replicates in which words were simulated to evolve through a process of gain and loss along the branches of a ClonalFrame phylogeny. The process of gain and loss was modelled so that the presence or absence of a word changed by any genetic mechanism on a branch with length *d* according to continuous‐time Markov chain with a probability of $1-\frac{\left(1+\exp\left(-2dr\right)\right)}{2}$, where *r* is rate (Sheppard *et al*., 2013a), and an inverse of total branch length was used. The null model assumes that the presence/absence of a word is randomly changed in the phylogeny irrespective of the biofilm formation. The null distribution will be normally distributed in the absence of phylogenetic population structure, while it will be a more complex distribution in the presence of population structure. Diversity within MLST genes of both datasets was similar (Fig. S6). Words with a *P*‐value below 0.001 were considered as targets for further examination and experimental testing with phenotyping assays. The distribution of words associated with biofilm production was mapped on the genome of *C. jejuni* NCTC11168 (Parkhill *et al*., 2000; Gundogdu *et al*., 2007) and visualized using Artemis (Carver *et al*., 2009). Words that did not map to *C. jejuni* NCTC11168 were located within isolates from the original dataset using BLAST. The open‐reading frames containing previously unmapped, biofilm‐associated words were submitted to the RAST annotation server to determine putative gene function.

### Verification GWAS

A second GWAS was performed on a smaller verification dataset. We tested sequenced isolates from the Swansea genome archive using the same biofilm protocol and grouped according to absorbance to form groups and subject to GWAS. The validation dataset included 13 isolates from ST‐21 and 14 isolates from ST‐45 that were grouped according to their biofilm readings (Table S1). As fewer isolates were used in the validation dataset, the association with biofilm phenotype was weaker and this is reflected in less stringent *P*‐values observed.

## Supporting information


**Fig. S1.** Distribution of biofilm absorbance readings grouped into upper (OD_600_ above 0.272), middle (OD_600_ between 0.201 and 0.272) or lower (OD_600_ below 0.201) 33rd percentiles. Red box plots indicate interquartile ranges.
**Fig. S2.** The null distributions of the association scores are shown for (A) ST‐21 and (B) ST‐45 clonal complexes. In ST‐21 clonal complex, a strong population structure is indicated by a bimodal distribution, with the most frequent association scores around −7 or 7. In ST‐45 clonal complex, a normal distribution indicates weak population structure. The dashed red line indicates cut‐off corresponding to *P* < 0.001 in each clonal complex. Distribution of *P*‐values for all observed words in (C) ST‐21 and (D) ST‐45 clonal complexes. Words are not uniformly distributed because many words tend to show the same *P*‐values.
**Fig. S3.** Growth of *Campylobacter* isolates during biofilm production under different O_2_ concentrations as measured by the change in absorbance (OD_600_). Dotted lines indicate standard errors. Growth under atmospheric (20%), 10% and 5% oxygen conditions are represented by black, red and blue lines respectively.
**Fig. S4.** The distribution of biofilm‐associated words identified by genome‐wide association studies in other clonal complexes. The proportion of ST‐21 and ST‐45 specific biofilm‐associated words is shown as a pie chart (red indicates the presence of the associated word, blue indicates absence of the associated word) alongside a neighbour joining tree of all isolates used in the study. Isolates on the tree are coloured by their ability to form biofilm: red for an OD_600_ above 0.272, pink for an OD_600_ between 0.201 and 0.272 and white for an OD_600_ below 0.201.
**Fig. S5.** Growth of *Campylobacter* isolates during biofilm production grouped by ecological groups as measured by the change in absorbance (OD_600_). Dotted lines indicate standard errors. Growth of host generalist (black), chicken specialist (orange), cattle specialist (blue) and *C. coli* (grey) is represented by coloured lines.
**Fig. S6.** Pairwise distance matrix between isolates used for (A) ST‐21 and (B) ST‐45 GWAS experiments. Values represent the number of loci with different allele sequences for the seven MLST genes (*aspA*, *glnA*, *gltA*, *glyA*, *pgm*, *tkt* and *uncA*). Isolate numbers and aliases are shown along both axes.
**Table S1.** List of isolates. Isolates highlighted in yellow were used in the original genome‐wide association studies.
**Table S2.** List of biofilm‐associated genes identified using the genome‐wide association studies. Grey shading indicates a hit in both the verification and original GWAS.
