# Supplementary material for: Enhanced biofilm formation and multi‐host transmission evolve from divergent genetic backgrounds in C ampylobacter jejuni
Source: Environ Microbiol. 2015 Oct 14;17(11):4779–89. doi: 10.1111/1462-2920.13051 (PMC4862030; doi:10.1111/1462-2920.13051)
Supplement: Supplementary file 1 — Fig. S1. Distribution of biofilm absorbance readings grouped into upper (OD600 above 0.272), middle (OD600 between 0.201 and 0.272) or lower (OD600 below 0.201) 33rd percentiles. Red box plots indicate interquartile ranges. Fig. S2. The null distributions of the association scores are shown for (A) ST‐21 and (B) ST‐45 clonal complexes. In ST‐21 clonal complex, a strong population structure is indicated by a bimodal distribution, with the most frequent association scores around −7 or 7. In ST‐45 clonal complex, a normal distribution indicates weak population structure. The dashed red line indicates cut‐off corresponding to P < 0.001 in each clonal complex. Distribution of P‐values for all observed words in (C) ST‐21 and (D) ST‐45 clonal complexes. Words are not uniformly distributed because many words tend to show the same P‐values. Fig. S3. Growth of Campylobacter isolates during biofilm production under different O2 concentrations as measured by the change in absorbance (OD600). Dotted lines indicate standard errors. Growth under atmospheric (20%), 10% and 5% oxygen conditions are represented by black, red and blue lines respectively. Fig. S4. The distribution of biofilm‐associated words identified by genome‐wide association studies in other clonal complexes. The proportion of ST‐21 and ST‐45 specific biofilm‐associated words is shown as a pie chart (red indicates the presence of the associated word, blue indicates absence of the associated word) alongside a neighbour joining tree of all isolates used in the study. Isolates on the tree are coloured by their ability to form biofilm: red for an OD600 above 0.272, pink for an OD600 between 0.201 and 0.272 and white for an OD600 below 0.201. Fig. S5. Growth of Campylobacter isolates during biofilm production grouped by ecological groups as measured by the change in absorbance (OD600). Dotted lines indicate standard errors. Growth of host generalist (black), chicken specialist (orange), cattle specialist (blue) and C. c [file EMI-17-4779-s001.zip › Figure S6.pdf]

A: ST-21

	36|CampsClin26	37|CampsClin88	46|cow2673	47|cow2674	59|cow518	60|CampsClin53	61|cow58	62|cowa21	63|chickc21	65|chick104	71|chick19	72|chick50	73|chick53	74|chick262	75|chick266	77|chick1086	78|chick1360	89|cowb21	93|cow53	94|cow104	97|cow3201	110|chickb21	113|chick883	116|CampsClin2	117|OxClina21	120|OxClinb45
36|CampsClin266	0																									
37|CampsClin883	3	0																								
46|cow2673	3	3	0																							
47|cow2674	2	2	1	0																						
59|cow518	2	2	1	0	0																					
60|CampsClin53	2	2	2	1	1	0																				
61|cow58	1	2	2	1	1	1	0																			
62|cowa21	2	2	1	0	0	1	1	0																		
63|chickc21	2	2	1	0	0	1	1	0	0																	
65|chick104	3	3	1	1	1	2	2	1	1	0																
71|chick19	2	2	2	1	1	1	1	1	1	2	0															
72|chick50	2	2	2	1	1	1	1	1	1	2	0	0														
73|chick53	2	2	2	1	1	0	1	1	1	2	1	1	0													
74|chick262	3	3	2	1	1	2	2	1	1	2	2	2	2	0												
75|chick266	0	3	3	2	2	2	1	2	2	3	2	2	2	3	0											
77|chick1086	2	2	2	1	1	1	1	1	1	2	0	0	1	2	2	0										
78|chick1360	2	2	2	1	1	1	1	1	1	2	0	0	1	2	2	0	0									
89|cowb21	2	2	1	0	0	1	1	0	0	1	1	1	1	1	2	1	1	0								
93|cow53	2	2	2	1	1	0	1	1	1	2	1	1	0	2	2	1	1	1	0							
94|cow104	3	3	1	1	1	2	2	1	1	0	2	2	2	2	3	2	2	1	2	0						
97|cow3201	1	2	2	1	1	1	0	1	1	2	1	1	1	2	1	1	1	1	1	2	0					
110|chickb21	2	2	1	0	0	1	1	0	0	1	1	1	1	1	2	1	1	0	1	1	1	0				
113|chick883	3	0	3	2	2	2	2	2	2	3	2	2	2	3	3	2	2	2	2	3	2	2	0			
116|CampsClin21	3	3	1	1	1	2	1	1	1	2	2	2	2	2	3	2	2	1	2	2	2	1	3	0		
117|OxClina21	2	2	1	0	0	1	1	0	0	1	1	1	1	1	2	1	1	0	1	1	1	0	2	1	0	
120|OxClinb45	2	2	1	0	0	1	1	0	0	1	1	1	1	1	2	1	1	0	1	1	1	0	2	1	0	0

B: ST-45

	4|CAMP45	32|CampsClin11	35|CampsClin583	38|CampsClin100	39|chick2219	45|chick594	52|cow334	55|CampsClin230	56|cowa45	57|chick2213	70|chickc45	79|chick11	81|chick1003	82|chick2048	84|chick2223	90|cowb45	91|cowc45	92|cowd45	100|cow137	101|cow230	102|cow583	103|cow3207	104|cow3214	111|chickb45	112|chickd45	118|OxClinb21	119|OxClina45	128|goose137	131|duck45
4|CAMP45	0																												
32|CampsClin11	1	0																											
35|CampsClin583	2	3	0																										
38|CampsClin1003	3	3	4	0																									
39|chick2219	1	1	3	3	0																								
45|chick594	2	3	0	4	3	0																							
52|cow334	2	3	2	3	3	2	0																						
55|CampsClin230	2	3	2	3	3	2	1	0																					
56|cowa45	0	1	2	3	1	2	2	2	0																				
57|chick2213	2	3	2	3	3	2	0	1	2	0																			
70|chickc45	0	1	2	3	1	2	2	2	0	2	0																		
79|chick11	1	0	3	3	1	3	3	3	1	3	1	0																	
81|chick1003	3	3	4	0	3	4	3	3	3	3	3	3	0																
82|chick2048	0	1	2	3	1	2	2	2	0	2	0	1	3	0															
84|chick2223	0	1	2	3	1	2	2	2	0	2	0	1	3	0	0														
90|cowb45	0	1	2	3	1	2	2	2	0	2	0	1	3	0	0	0													
91|cowc45	0	1	2	3	1	2	2	2	0	2	0	1	3	0	0	0	0												
92|cowd45	0	1	2	3	1	2	2	2	0	2	0	1	3	0	0	0	0	0											
100|cow137	1	2	1	3	2	1	1	1	1	1	1	2	3	1	1	1	1	1	0										
101|cow230	2	2	2	2	2	2	1	0	2	1	2	2	2	2	2	2	2	2	1	0									
102|cow583	2	3	0	4	3	0	2	2	2	2	2	3	4	2	2	2	2	2	1	2	0								
103|cow3207	2	3	2	3	3	2	0	1	2	0	2	3	3	2	2	2	2	2	1	1	2	0							
104|cow3214	0	1	2	3	1	2	2	2	0	2	0	1	3	0	0	0	0	0	1	2	2	2	0						
111|chickb45	0	1	2	3	1	2	2	2	0	2	0	1	3	0	0	0	0	0	1	2	2	2	0	0					
112|chickd45	0	1	2	3	1	2	2	2	0	2	0	1	3	0	0	0	0	0	1	2	2	2	0	0	0				
118|OxClinb21	0	1	2	3	1	2	2	2	0	2	0	1	3	0	0	0	0	0	1	2	2	2	0	0	0	0			
119|OxClina45	0	1	2	3	1	2	2	2	0	2	0	1	3	0	0	0	0	0	1	2	2	2	0	0	0	0	0		
128|goose137	1	2	1	3	2	1	1	1	1	1	1	2	3	1	1	1	1	1	0	1	1	1	1	1	1	1	1	0	
131|duck45	0	1	2	3	1	2	2	2	0	2	0	1	3	0	0	0	0	0	1	2	2	2	0	0	0	0	0	1	0

Distances between isolates calculated as the number of loci with different allele sequences for 7 MLST genes.
