# Supplementary material for: Enhanced biofilm formation and multi‐host transmission evolve from divergent genetic backgrounds in C ampylobacter jejuni
Source: Environ Microbiol. 2015 Oct 14;17(11):4779–89. doi: 10.1111/1462-2920.13051 (PMC4862030; doi:10.1111/1462-2920.13051)
Supplement: Supplementary file 1 — Fig. S1. Distribution of biofilm absorbance readings grouped into upper (OD600 above 0.272), middle (OD600 between 0.201 and 0.272) or lower (OD600 below 0.201) 33rd percentiles. Red box plots indicate interquartile ranges. Fig. S2. The null distributions of the association scores are shown for (A) ST‐21 and (B) ST‐45 clonal complexes. In ST‐21 clonal complex, a strong population structure is indicated by a bimodal distribution, with the most frequent association scores around −7 or 7. In ST‐45 clonal complex, a normal distribution indicates weak population structure. The dashed red line indicates cut‐off corresponding to P < 0.001 in each clonal complex. Distribution of P‐values for all observed words in (C) ST‐21 and (D) ST‐45 clonal complexes. Words are not uniformly distributed because many words tend to show the same P‐values. Fig. S3. Growth of Campylobacter isolates during biofilm production under different O2 concentrations as measured by the change in absorbance (OD600). Dotted lines indicate standard errors. Growth under atmospheric (20%), 10% and 5% oxygen conditions are represented by black, red and blue lines respectively. Fig. S4. The distribution of biofilm‐associated words identified by genome‐wide association studies in other clonal complexes. The proportion of ST‐21 and ST‐45 specific biofilm‐associated words is shown as a pie chart (red indicates the presence of the associated word, blue indicates absence of the associated word) alongside a neighbour joining tree of all isolates used in the study. Isolates on the tree are coloured by their ability to form biofilm: red for an OD600 above 0.272, pink for an OD600 between 0.201 and 0.272 and white for an OD600 below 0.201. Fig. S5. Growth of Campylobacter isolates during biofilm production grouped by ecological groups as measured by the change in absorbance (OD600). Dotted lines indicate standard errors. Growth of host generalist (black), chicken specialist (orange), cattle specialist (blue) and C. c [file EMI-17-4779-s001.zip › Table S1.pdf]

Isolate ID	Isolate	Source	Clonal Complex	Biofilm	Biofilm 33rd percentile	Ref
4	CAMP45	chicken	ST-45	0.161	Lower	1
5	CAMP2696	pig	--	0.268	Middle	1
6	CAMP2681	chicken	--	0.193	Lower	1
7	CAMP1576	chicken	--	0.255	Middle	1
8	CAMP1670	human	--	0.267	Middle	1
9	CAMP1643	human	--	0.335	Upper	1
10	CAMP1572	chicken	--	0.205	Middle	1
11	CAMP2016	duck	--	0.217	Middle	1
12	CAMP2326	--	--	0.238	Middle	1
13	CAMP61	cattle	ST-61	0.290	Upper	1
15	CAMP886	pig	ST-828	0.230	Middle	1
16	CAMP1771	duck	--	0.213	Middle	1
18	CAMP3667	chicken	ST-1150	0.218	Middle	1
19	CAMP3129	--	--	0.374	Upper	1
20	CAMP1487	chicken	--	0.272	Upper	1
21	CAMP1090	chicken	ST-828	0.265	Middle	1
22	CAMP2488	chicken	ST-257	0.244	Middle	1
23	CAMP3311	duck	--	0.229	Middle	1
24	CAMP828	chicken	ST-828	0.272	Upper	1
25	CAMP3136	--	--	0.277	Upper	1
32	CampsClin11	human	ST-45	0.176	Lower	2
35	CampsClin583	human	ST-45	0.423	Upper	2
36	CampsClin266	human	ST-21	0.260	Middle	2
37	CampsClin883	human	ST-21	0.277	Upper	2
38	CampsClin1003	human	ST-45	0.291	Upper	2
39	chick2219	chicken	ST-45	0.317	Upper	2
42	cow42	cattle	ST-42	0.224	Middle	2
43	chick2253	chicken	--	0.162	Lower	2
44	chick1717	chicken	--	0.186	Lower	2
45	chick594	chicken	ST-45	0.261	Middle	2
46	cow2673	cattle	ST-21	0.176	Lower	2

Isolate ID	Isolate	Source	Clonal Complex	Biofilm	Biofilm 33rd percentile	Ref
47	cow2674	cattle	ST-21	0.242	Middle	2
48	cow206	cattle	ST-206	0.173	Lower	2
49	cow38	cattle	ST-48	0.214	Middle	2
50	cow190	cattle	--	0.217	Middle	2
52	cow334	cattle	ST-45	0.400	Upper	2
54	chick267	chicken	ST-283	0.242	Middle	2
55	CampsClin230	human	ST-45	0.265	Middle	2
56	cowa45	cattle	ST-45	0.261	Middle	2
57	chick2213	chicken	ST-45	0.258	Middle	2
59	cow518	cattle	ST-21	0.523	Upper	2
60	CampsClin53	human	ST-21	0.256	Middle	2
61	cow58	cattle	ST-21	0.302	Upper	2
62	cowa21	cattle	ST-21	0.404	Upper	2
63	chickc21	chicken	ST-21	0.559	Upper	2
64	chick25	chicken	ST-661	0.191	Lower	2
65	chick104	chicken	ST-21	0.112	Lower	2
66	chick353	chicken	ST-353	0.177	Lower	2
67	chickb354	chicken	ST-354	0.160	Lower	2
68	chick573	chicken	ST-573	0.279	Upper	2
69	chick2568	chicken	ST-661	0.228	Middle	2
70	chickc45	chicken	ST-45	0.303	Upper	2
71	chick19	chicken	ST-21	0.501	Upper	2
72	chick50	chicken	ST-21	0.289	Upper	2
73	chick53	chicken	ST-21	0.197	Lower	2
74	chick262	chicken	ST-21	0.268	Middle	2
75	chick266	chicken	ST-21	0.177	Lower	2
77	chick1086	chicken	ST-21	0.349	Upper	2
78	chick1360	chicken	ST-21	0.301	Upper	2
79	chick11	chicken	ST-45	0.384	Upper	2
80	chick137	chicken	ST-257	0.214	Middle	2
81	chick1003	chicken	ST-45	0.201	Lower	2

Isolate ID	Isolate	Source	Clonal Complex	Biofilm	Biofilm 33rd percentile	Ref
82	chick2048	chicken	ST-45	0.176	Lower	2
83	chick2197	chicken	ST-354	0.174	Lower	2
84	chick2223	chicken	ST-45	0.201	Middle	2
85	cow3583	cattle	ST-42	0.194	Lower	2
86	cow618	cattle	ST-61	0.208	Middle	2
87	cow273	cattle	ST-206	0.278	Upper	2
89	cowb21	cattle	ST-21	0.280	Upper	2
90	cowb45	cattle	ST-45	0.153	Lower	2
91	cowc45	cattle	ST-45	0.270	Middle	2
92	cowd45	cattle	ST-45	0.127	Lower	2
93	cow53	cattle	ST-21	0.537	Upper	2
94	cow104	cattle	ST-21	0.103	Lower	2
96	cow3189	cattle		0.192	Lower	2
97	cow3201	cattle	ST-21	0.163	Lower	2
98	cow3202	cattle	ST-828	0.231	Middle	2
99	cow3205	cattle	ST-206	0.072	Lower	2
100	cow137	cattle	ST-45	0.308	Upper	2
101	cow230	cattle	ST-45	0.383	Upper	2
102	cow583	cattle	ST-45	0.217	Middle	2
103	cow3207	cattle	ST-45	0.603	Upper	2
104	cow3214	cattle	ST-45	0.080	Lower	2
105	chick354	chicken	ST-257	0.066	Lower	2
106	chick51	chicken	ST-443	0.082	Lower	2
107	chick1079	chicken	ST-573	0.154	Lower	2
108	chick574	chicken	ST-574	0.758	Upper	2
109	chick814	chicken	ST-661	0.096	Lower	2
110	chickb21	chicken	ST-21	0.232	Middle	2
111	chickb45	chicken	ST-45	0.087	Lower	2
112	chickd45	chicken	ST-45	0.087	Lower	2
113	chick883	chicken	ST-21	0.213	Middle	2
116	CampsClin21	human	ST-21	0.153	Lower	2

Isolate ID	Isolate	Source	Clonal Complex	Biofilm	Biofilm 33rd percentile	Ref
117	OxClina21	human	ST-21	0.237	Middle	2
118	OxClinb21	human	ST-45	0.274	Upper	2
119	OxClina45	human	ST-45	0.118	Lower	2
120	OxClinb45	human	ST-21	0.729	Upper	2
122	starling177	starling	ST-177	0.134	Lower	2
123	starling682	starling	ST-682	0.274	Upper	2
125	starling1020	starling	ST-682	0.620	Upper	2
128	goose137	goose	ST-45	0.109	Lower	2
131	duck45	duck	ST-45	1.005	Upper	2
342	SS_091	chicken	ST-45	0.233	verification	3
371	SS_169	chicken	ST-45	0.192	verification	3
384	SS_187	chicken	ST-21	0.116	verification	3
392	SS_218	chicken	ST-45	0.143	verification	3
405	SS_252	chicken	ST-45	0.271	verification	3
418	SS_297	chicken	ST-21	0.149	verification	3
421	SS_301	chicken	ST-21	1.231	verification	3
423	SS_303	chicken	ST-21	0.155	verification	3
489	SS_108	farm	ST-21	0.109	verification	3
490	SS_109	farm	ST-21	0.113	verification	3
1702	SS_140	farm	ST-45	0.211	verification	3
1714	SS_181	farm	ST-21	0.846	verification	3
1722	SS_197	farm	ST-45	0.193	verification	3
1731	SS_222	farm	ST-21	0.478	verification	3
1733	SS_230	farm	ST-45	0.163	verification	3
1737	SS_237	farm	ST-45	0.468	verification	3
1738	SS_238	farm	ST-45	0.142	verification	3
1740	SS_244	farm	ST-45	0.145	verification	3
1751	SS_271	human	ST-45	0.183	verification	3
1800	SS_375	chicken	ST-45	0.132	verification	3
1863	OXC5333	human	ST-21	0.201	verification	3
1865	OXC5335	human	ST-21	0.462	verification	3

Isolate ID	Isolate	Source	Clonal Complex	Biofilm	Biofilm 33rd percentile	Ref
1881	OXC5364	human	ST-21	0.123	verification	3
1932	OXC5691	human	ST-21	0.248	verification	3
2015	OXC5850	human	ST-45	0.246	verification	3
2195	OXC5832	human	ST-21	0.134	verification	3
2241	OXC5925	human	ST-21	0.273	verification	3

[1]Sheppard et al 2013a
[2]Sheppard et al 2013b
[3]Unpublished
