# Supplementary material for: Enhanced biofilm formation and multi‐host transmission evolve from divergent genetic backgrounds in C ampylobacter jejuni
Source: Environ Microbiol. 2015 Oct 14;17(11):4779–89. doi: 10.1111/1462-2920.13051 (PMC4862030; doi:10.1111/1462-2920.13051)
Supplement: Supplementary file 1 — Fig. S1. Distribution of biofilm absorbance readings grouped into upper (OD600 above 0.272), middle (OD600 between 0.201 and 0.272) or lower (OD600 below 0.201) 33rd percentiles. Red box plots indicate interquartile ranges. Fig. S2. The null distributions of the association scores are shown for (A) ST‐21 and (B) ST‐45 clonal complexes. In ST‐21 clonal complex, a strong population structure is indicated by a bimodal distribution, with the most frequent association scores around −7 or 7. In ST‐45 clonal complex, a normal distribution indicates weak population structure. The dashed red line indicates cut‐off corresponding to P < 0.001 in each clonal complex. Distribution of P‐values for all observed words in (C) ST‐21 and (D) ST‐45 clonal complexes. Words are not uniformly distributed because many words tend to show the same P‐values. Fig. S3. Growth of Campylobacter isolates during biofilm production under different O2 concentrations as measured by the change in absorbance (OD600). Dotted lines indicate standard errors. Growth under atmospheric (20%), 10% and 5% oxygen conditions are represented by black, red and blue lines respectively. Fig. S4. The distribution of biofilm‐associated words identified by genome‐wide association studies in other clonal complexes. The proportion of ST‐21 and ST‐45 specific biofilm‐associated words is shown as a pie chart (red indicates the presence of the associated word, blue indicates absence of the associated word) alongside a neighbour joining tree of all isolates used in the study. Isolates on the tree are coloured by their ability to form biofilm: red for an OD600 above 0.272, pink for an OD600 between 0.201 and 0.272 and white for an OD600 below 0.201. Fig. S5. Growth of Campylobacter isolates during biofilm production grouped by ecological groups as measured by the change in absorbance (OD600). Dotted lines indicate standard errors. Growth of host generalist (black), chicken specialist (orange), cattle specialist (blue) and C. c [file EMI-17-4779-s001.zip › Table S2_new.pdf]

Gene identifier	11168 nomenclature	Alias	Number of associated WORDS	Transcriptional unit number (according to OperonPredictor1)	RAST description	Proposed function	GWAS	Position start	Position end
CAMP0020	Cj0020c		30	13	cytochrome C551 peroxidase (EC:1.11.1.5)		ST-21	25,433	26,347
CAMP0119	Cj0131		42	54	Similarity to metalloendopeptidases (cell wall biosynthesis)	cell wall biosynthesis	ST-45	133,003	134,376
CAMP0122	Cj0134	*thrB*	54	54	Homoserine kinase (EC 2.7.1.39)		ST-45	135,709	136,587
CAMP0123	Cj0135		20	54	Hypothetical protein Cj0135		ST-45	136,612	136,869
CAMP0124	Cj0136	*infB*	327	54	Translation initiation factor 2		ST-45	136,856	139,471
CAMP0125	Cj0137	*rbfA*	32	54	ribosome-binding factor A		ST-45	139,468	139,830
CAMP0126	Cj0138		15	54	FIG000325: clustered with transcription termination protein NusA		ST-45	139,820	140,242
CAMP0134	Cj0146c	*trxB*	36	58	Thioredoxin reductase (EC 1.8.1.9)	Sensing Oxidative Stress	ST-45	150,638	151,576
CAMP0135	Cj0147c	*trxA*	7	58	Thioredoxin		ST-45	151,710	152,024
CAMP0137	Cj0149c	*hom*	73	58	Homoserine dehydrogenase (EC 1.1.1.3)		ST-45	152,419	153,666
CAMP0138	Cj0150c		44	58	Aspartate aminotransferase (EC 2.6.1.1)		ST-45	153,670	154,872
CAMP0139	Cj0151c		30	59	membrane protein	Metal uptake	ST-45	154,884	155,690
CAMP0146	Cj0158c		49	62	Queuosine biosynthesis QueD, PTPS-I		ST-45	159,483	159,908
CAMP0147	Cj0159c		60	63	Queuosine Biosynthesis QueE Radical SAM		ST-45	159,908	160,489
CAMP0189	Cj0203		2	80	Magnesium citrate secondary transporter		ST-45	197,707	199,053
CAMP0233	Cj0263	*zupT*	1	103	Zinc transporter ZupT		ST-45	241,022	241,897
CAMP0237	Cj0267c		42	105	membrane protein	Sensing Oxidative Stress	ST-45	246,013	246,543

Gene identifier	11168 nomenclature	Alias	Number of associated WORDS	Transcriptional unit number (according to OperonPredictor1)	RAST description	Proposed function	GWAS	Position start	Position end
CAMP0238	Cj0268c		12	105	Membrane protease family protein HP0248		ST-45	246,555	247,643
CAMP0239	Cj0269c	*ilvE*	57	105	Branched-chain amino acid aminotransferase (EC 2.6.1.42)		ST-45	247,656	248,570
CAMP0241	Cj0271		71	106	Thiol peroxidase, Bcp-type (EC 1.11.1.15)	Iron uptake	ST-45	248,950	249,405
CAMP0242	Cj0272		3	106	FIG053235: Diacylglucosamine hydrolase like		ST-45	249,405	250,496
CAMP0243	Cj0273	*fabZ*	30	107	(3R)-hydroxymyristoyl-[acyl carrier protein] dehydratase (EC 4.2.1.-)	cell wall biosynthesis	ST-45	250,590	251,030
CAMP0244	Cj0274	*lpxA*	101	107	Acyl-[acyl-carrier-protein]--UDP-N-acetylglucosamine O-acyltransferase (EC 2.3.1.129)		ST-45	251,030	251,821
CAMP0248	Cj0279	*carB*	18	108	Carbamoyl-phosphate synthase large chain (EC 6.3.5.5)		ST-45	255,089	258,358
CAMP0250	Cj0281c	*tal*	9	110	Phosphoserine phosphatase (EC 3.1.3.3)	Chemotaxis	ST-45	258,778	259,755
CAMP0251	Cj0282c	*serB*	31	110	Positive regulator of CheA protein activity (CheW)		ST-45	259,755	260,378
CAMP0253	Cj0284c	*cheA*	73	110	Signal transduction histidine kinase CheA (EC 2.7.3.-)		ST-45	260,904	263,213
CAMP0270	Cj0304c	*bioC*	21	116	Biotin synthesis protein BioG	Biotin synthesis (Vitamin B7)	ST-45	276,925	277,611
CAMP0339	Cj0374		11	146	hypothetical protein		ST-21	342,115	342,606
CAMP0478	Cj0518	*htpG*	23	206	Chaperone protein HtpG	Heat shock protein	ST-45	483,003	484,829
CAMP0976	Cj1053c		9	396	UDP-N-acetylmuramate--alanine ligase (EC 6.3.2.8)	cell wall biosynthesis	ST-45	989,219	989,569

Gene identifier	11168 nomenclature	Alias	Number of associated WORDS	Transcriptional unit number (according to OperonPredictor1)	RAST description	Proposed function	GWAS	Position start	Position end
CAMP1200	Cj1282	*mrdB*	30	483	Rod shape-determining protein RodA	cell wall biosynthesis	ST-45	1,213,903	1,215,003
CAMP1224	Cj1306c		6	490	NADH dehydrogenase subunit C(EC:1.6.5.3)	Glycosylation locus (CJ1293-1342)	ST-45	1,235,522	1,236,748
CAMP1228	Cj1310c		6	492	putative peptide ABC-transport system ATP-binding protein		ST-45	1,239,673	1,240,887
CAMP1258	Cj1342c	*maf7*	15	500	putative peptide ABC-transport system permease protein		ST-45	1,274,694	1,275,935
CAMP1259	Cj1343c		12	500	putative bacterial haemoglobin		ST-45	1,275,935	1,276,450
CAMP1322	Cj1411c		9	528	cytochrome p450		ST-45	1,342,550	1,343,911
CAMP1356	Cj1445c	*kpsE*	28	532	Capsular polysaccharide ABC transporter, ATP-binding protein KpsT	Capsule	ST-45	1,385,146	1,386,264
CAMP1357	Cj1447c	*kpsT*	21	532	Capsular polysaccharide ABC transporter, permease protein KpsM		ST-45	1,386,264	1,386,926
CAMP1358	Cj1448c	*kpsM*	14	532	Capsular polysaccharide ABC transporter, permease protein KpsM		ST-45	1,386,923	1,387,705
CAMP1360	Cj1450		72	534	Putative ATP/GTP-binding protein (regulated by FliA)	motility	ST-45	1,388,259	1,388,822
CAMP1473	Cj1577c	*nuoC*	23	578	NADH-ubiquinone oxidoreductase chain B (EC 1.6.5.3)	Sensing Oxidative Stress (An/aerobic switch)	ST-45	1,508,131	1,508,925
CAMP1477	Cj1581c	*nikW*	30	579	Putative peptide ABC-transport system permease protein	Nickel transport (nikZYXWV)	ST-45	1,510,567	1,511,277

Gene identifier	11168 nomenclature	Alias	Number of associated WORDS	Transcriptional unit number (according to OperonPredictor1)	RAST description	Proposed function	GWAS	Position start	Position end
CAMP1479	Cj1583c	*nikY*	21	579	Oligopeptide ABC transporter, periplasmic oligopeptide-binding protein OppA (TC 3.A.1.5.1)	Nickel transport (nikZYXWV)	ST-45	1,512,055	1,512,999
CAMP1482	Cj1586	*cgb*	30	580	single domain hemoglobin	Nitrosative stress	ST-45	1,517,567	1,517,989
CAMP1622	Cj1729c	*flgE2*	7	634	flagellar hook protein	Flagella	ST-21	1,638,104	1,640,701
unmapped			5		Na+/H+ antiporter		ST-21		
unmapped			4		putative molybdenum containing oxidoreductase		ST-21		
unmapped			3		Ferric receptor CfrA		ST-45		
unmapped			12		Translation initiation factor 2		ST-45		
unmapped			1		FIG00711847: hypothetical protein		ST-45		
unmapped			9		FKBP-type peptidyl-prolyl cis-trans isomerase SlyD (EC 5.2.1.8)		ST-45		
unmapped			2		DNA-directed RNA polymerase alpha subunit (EC 2.7.7.6)		ST-45		
unmapped			5		Leucyl-tRNA synthetase (EC 6.1.1.4)		ST-45		
unmapped			15		Arginine/ornithine antiporter ArcD		ST-45		
unmapped			6		Similarity with glutathionylspermidine synthase (EC 6.3.1.8), group 2		ST-45		
unmapped			3		DNA-directed RNA polymerase beta' subunit (EC 2.7.7.6)		ST-45		
unmapped			9		Cell division protein FtsK		ST-45		

Gene identifier	11168 nomenclature	Alias	Number of associated WORDS	Transcriptional unit number (according to OperonPredictor1)	RAST description	Proposed function	GWAS	Position start	Position end
unmapped			11		Putative periplasmic ATP/GTP-binding protein		ST-45		
unmapped			1		Putative transcriptional regulator		ST-45		
unmapped			15		Diaminopimelate decarboxylase (EC 4.1.1.20)		ST-45		
unmapped			8		Thioredoxin reductase (EC 1.8.1.9)		ST-45		
unmapped			3		FIG00545237: hypothetical protein		ST-45		
unmapped			3		Outer membrane liproprotein mapA precursor		ST-45		
unmapped			1		Phospholipase A1 precursor (EC 3.1.1.32, EC 3.1.1.4); Outer membrane phospholipase A		ST-45		
unmapped			11		Phosphoenolpyruvate synthase / Pyruvate phosphate dikinase		ST-45		
unmapped			6		tRNA (uracil(54)-C5)-methyltransferase (EC 2.1.1.35)		ST-45		
unmapped			3		Cysteine desulfurase (EC 2.8.1.7)		ST-45		
unmapped			3		RNA polymerase sigma factor RpoD		ST-45		
unmapped			2		FIG00469724: hypothetical protein		ST-45		
unmapped			5		Type I restriction-modification system, DNA-methyltransferase subunit M (EC 2.1.1.72) / Type I restriction-modification system, specificity subunit S (EC 3.1.21.3)		ST-45		

Gene identifier	11168 nomenclature	Alias	Number of associated WORDS	Transcriptional unit number (according to OperonPredictor1)	RAST description	Proposed function	GWAS	Position start	Position end
unmapped			6		4-hydroxy-tetrahydrodipicolinate synthase (EC 4.3.3.7)		ST-45		
unmapped			2		Branched-chain amino acid transport system permease protein LivM (TC 3.A.1.4.1)		ST-45		
unmapped			1		hypothetical protein		ST-45		
unmapped			1		Octaprenyl diphosphate synthase (EC 2.5.1.90) / Dimethylallyltransferase (EC 2.5.1.1) / (2E,6E)-farnesyl diphosphate synthase (EC 2.5.1.10) / Geranylgeranyl diphosphate synthase (EC 2.5.1.29)		ST-45		
unmapped			11		Aminoacyl-histidine dipeptidase (Peptidase D) (EC 3.4.13.3)		ST-45		
unmapped			9		Putative iron-uptake ABC transport system ATP-binding protein		ST-45		
unmapped			16		Proline dehydrogenase (EC 1.5.99.8) (Proline oxidase) / Delta-1-pyrroline-5-carboxylate dehydrogenase (EC 1.2.1.88)		ST-45		
unmapped			3		Iron-sulfur cluster assembly scaffold protein IscU/NifU-like		ST-45		
unmapped			6		Putative transmembrane transport protein		ST-45		
unmapped			8		LSU m3Psi1915 methyltransferase RlmH		ST-45		

Gene identifier	11168 nomenclature	Alias	Number of associated WORDS	Transcriptional unit number (according to OperonPredictor1)	RAST description	Proposed function	GWAS	Position start	Position end
unmapped			2		DNA-directed RNA polymerase beta' subunit (EC 2.7.7.6)		ST-45		
unmapped			1		Signal transduction histidine kinase CheA (EC 2.7.3.-)		ST-45		
unmapped			3		Selenocysteine-specific translation elongation factor		ST-45		
unmapped			3		Aspartokinase (EC 2.7.2.4)		ST-45		
unmapped			1		Capsular polysaccharide biosynthesis heptosyltransferase HddD		ST-45		
unmapped			2		Signal recognition particle, subunit Ffh SRP54 (TC 3.A.5.1.1)		ST-45		
unmapped			2		hypothetical protein		ST-45		
unmapped			1		Lipopolysaccharide biosynthesis protein RffA		ST-45		

[1]Taboada *et al.*, 2012
