# Supplementary figures and images for: Enhanced biofilm formation and multi‐host transmission evolve from divergent genetic backgrounds in C ampylobacter jejuni
Source: Environ Microbiol. 2015 Oct 14;17(11):4779–89. doi: 10.1111/1462-2920.13051 (PMC4862030; doi:10.1111/1462-2920.13051)

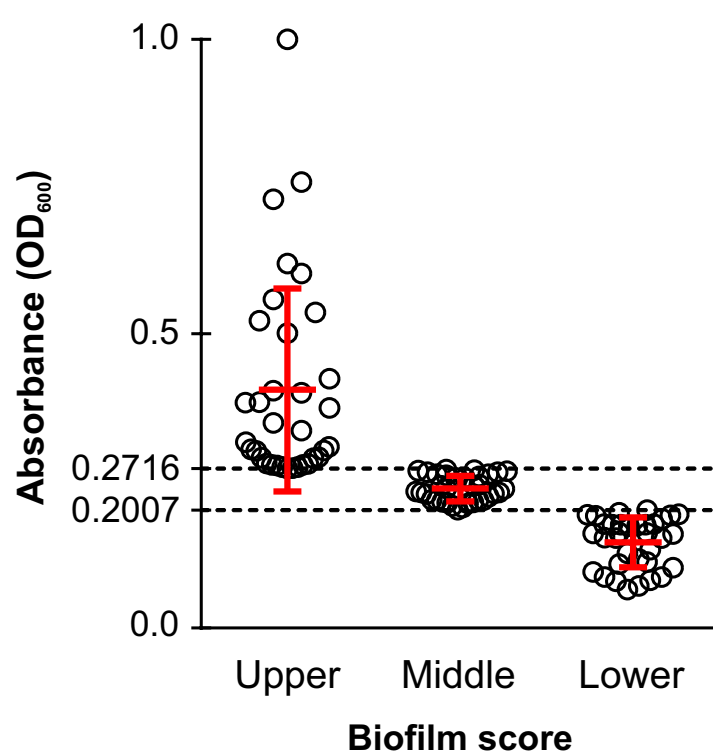

Supplement: Supplementary file 1 — Fig. S1. Distribution of biofilm absorbance readings grouped into upper (OD600 above 0.272), middle (OD600 between 0.201 and 0.272) or lower (OD600 below 0.201) 33rd percentiles. Red box plots indicate interquartile ranges. Fig. S2. The null distributions of the association scores are shown for (A) ST‐21 and (B) ST‐45 clonal complexes. In ST‐21 clonal complex, a strong population structure is indicated by a bimodal distribution, with the most frequent association scores around −7 or 7. In ST‐45 clonal complex, a normal distribution indicates weak population structure. The dashed red line indicates cut‐off corresponding to P < 0.001 in each clonal complex. Distribution of P‐values for all observed words in (C) ST‐21 and (D) ST‐45 clonal complexes. Words are not uniformly distributed because many words tend to show the same P‐values. Fig. S3. Growth of Campylobacter isolates during biofilm production under different O2 concentrations as measured by the change in absorbance (OD600). Dotted lines indicate standard errors. Growth under atmospheric (20%), 10% and 5% oxygen conditions are represented by black, red and blue lines respectively. Fig. S4. The distribution of biofilm‐associated words identified by genome‐wide association studies in other clonal complexes. The proportion of ST‐21 and ST‐45 specific biofilm‐associated words is shown as a pie chart (red indicates the presence of the associated word, blue indicates absence of the associated word) alongside a neighbour joining tree of all isolates used in the study. Isolates on the tree are coloured by their ability to form biofilm: red for an OD600 above 0.272, pink for an OD600 between 0.201 and 0.272 and white for an OD600 below 0.201. Fig. S5. Growth of Campylobacter isolates during biofilm production grouped by ecological groups as measured by the change in absorbance (OD600). Dotted lines indicate standard errors. Growth of host generalist (black), chicken specialist (orange), cattle specialist (blue) and C. c [file EMI-17-4779-s001.zip › Figure S1.pdf]

**A: ST-21**

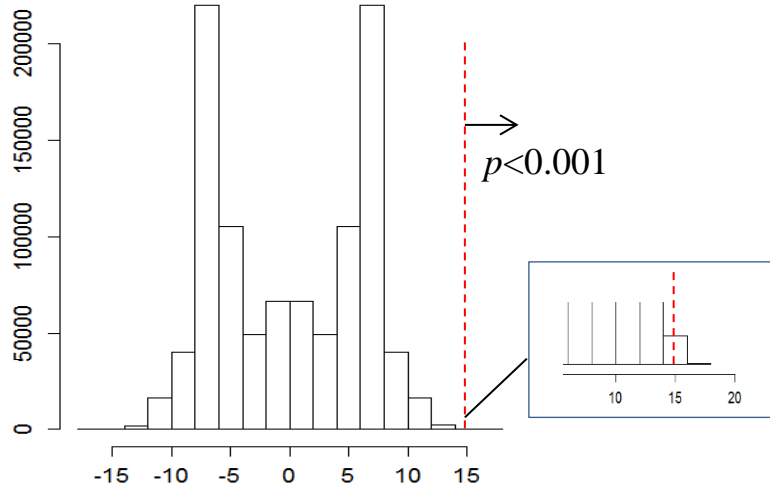

association score (null)

**B: ST-45**

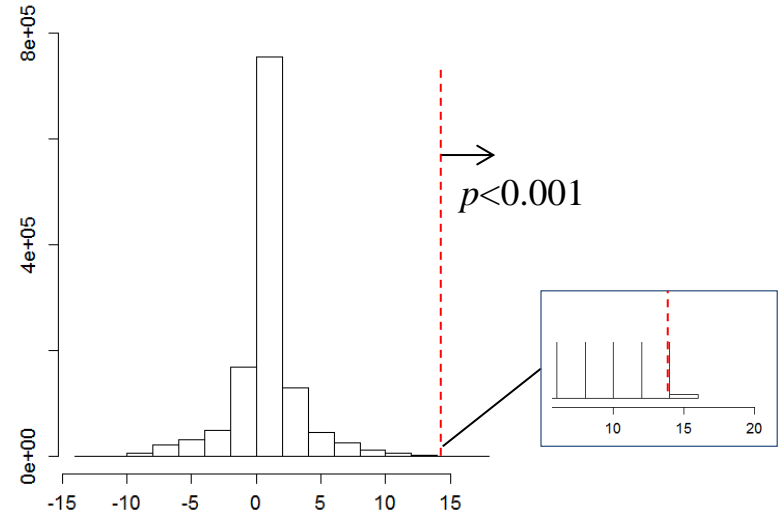

association score (null)

**C: ST-21**

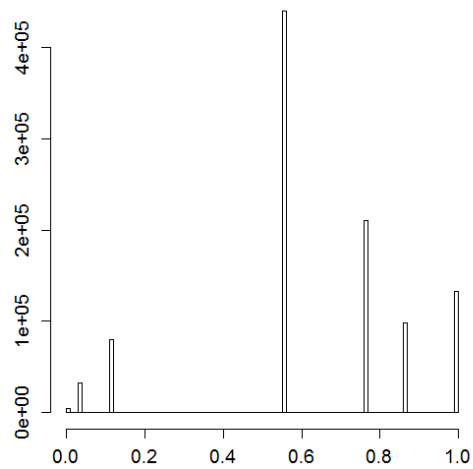

P value

**D: ST-45**

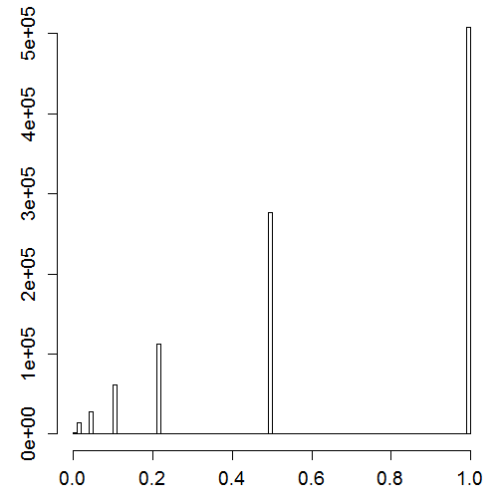

P value

Supplement: Supplementary file 1 — Fig. S1. Distribution of biofilm absorbance readings grouped into upper (OD600 above 0.272), middle (OD600 between 0.201 and 0.272) or lower (OD600 below 0.201) 33rd percentiles. Red box plots indicate interquartile ranges. Fig. S2. The null distributions of the association scores are shown for (A) ST‐21 and (B) ST‐45 clonal complexes. In ST‐21 clonal complex, a strong population structure is indicated by a bimodal distribution, with the most frequent association scores around −7 or 7. In ST‐45 clonal complex, a normal distribution indicates weak population structure. The dashed red line indicates cut‐off corresponding to P < 0.001 in each clonal complex. Distribution of P‐values for all observed words in (C) ST‐21 and (D) ST‐45 clonal complexes. Words are not uniformly distributed because many words tend to show the same P‐values. Fig. S3. Growth of Campylobacter isolates during biofilm production under different O2 concentrations as measured by the change in absorbance (OD600). Dotted lines indicate standard errors. Growth under atmospheric (20%), 10% and 5% oxygen conditions are represented by black, red and blue lines respectively. Fig. S4. The distribution of biofilm‐associated words identified by genome‐wide association studies in other clonal complexes. The proportion of ST‐21 and ST‐45 specific biofilm‐associated words is shown as a pie chart (red indicates the presence of the associated word, blue indicates absence of the associated word) alongside a neighbour joining tree of all isolates used in the study. Isolates on the tree are coloured by their ability to form biofilm: red for an OD600 above 0.272, pink for an OD600 between 0.201 and 0.272 and white for an OD600 below 0.201. Fig. S5. Growth of Campylobacter isolates during biofilm production grouped by ecological groups as measured by the change in absorbance (OD600). Dotted lines indicate standard errors. Growth of host generalist (black), chicken specialist (orange), cattle specialist (blue) and C. c [file EMI-17-4779-s001.zip › Figure S2_new.pdf]

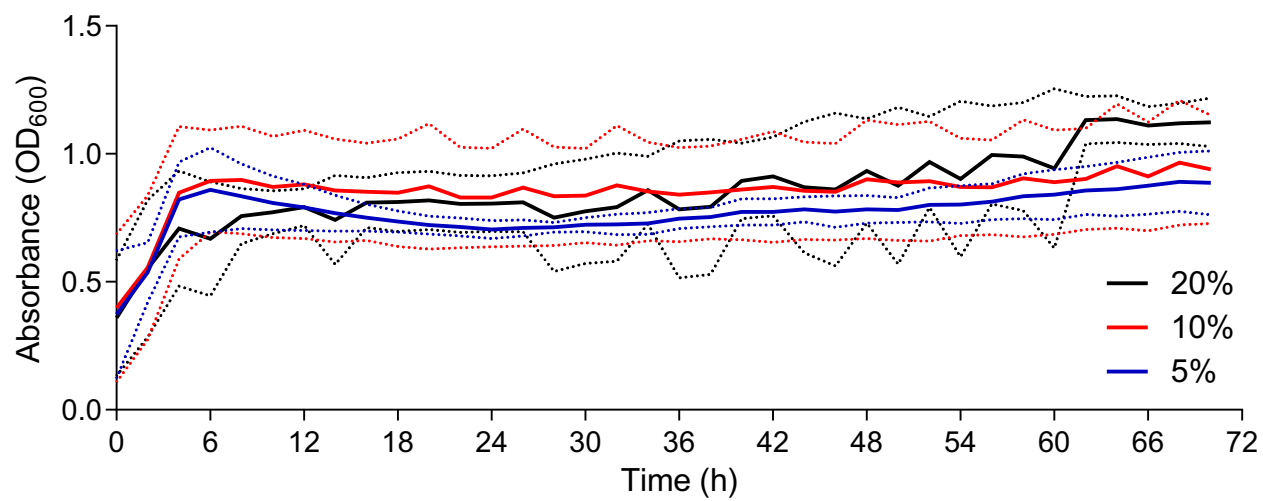

Supplement: Supplementary file 1 — Fig. S1. Distribution of biofilm absorbance readings grouped into upper (OD600 above 0.272), middle (OD600 between 0.201 and 0.272) or lower (OD600 below 0.201) 33rd percentiles. Red box plots indicate interquartile ranges. Fig. S2. The null distributions of the association scores are shown for (A) ST‐21 and (B) ST‐45 clonal complexes. In ST‐21 clonal complex, a strong population structure is indicated by a bimodal distribution, with the most frequent association scores around −7 or 7. In ST‐45 clonal complex, a normal distribution indicates weak population structure. The dashed red line indicates cut‐off corresponding to P < 0.001 in each clonal complex. Distribution of P‐values for all observed words in (C) ST‐21 and (D) ST‐45 clonal complexes. Words are not uniformly distributed because many words tend to show the same P‐values. Fig. S3. Growth of Campylobacter isolates during biofilm production under different O2 concentrations as measured by the change in absorbance (OD600). Dotted lines indicate standard errors. Growth under atmospheric (20%), 10% and 5% oxygen conditions are represented by black, red and blue lines respectively. Fig. S4. The distribution of biofilm‐associated words identified by genome‐wide association studies in other clonal complexes. The proportion of ST‐21 and ST‐45 specific biofilm‐associated words is shown as a pie chart (red indicates the presence of the associated word, blue indicates absence of the associated word) alongside a neighbour joining tree of all isolates used in the study. Isolates on the tree are coloured by their ability to form biofilm: red for an OD600 above 0.272, pink for an OD600 between 0.201 and 0.272 and white for an OD600 below 0.201. Fig. S5. Growth of Campylobacter isolates during biofilm production grouped by ecological groups as measured by the change in absorbance (OD600). Dotted lines indicate standard errors. Growth of host generalist (black), chicken specialist (orange), cattle specialist (blue) and C. c [file EMI-17-4779-s001.zip › Figure S3.pdf]

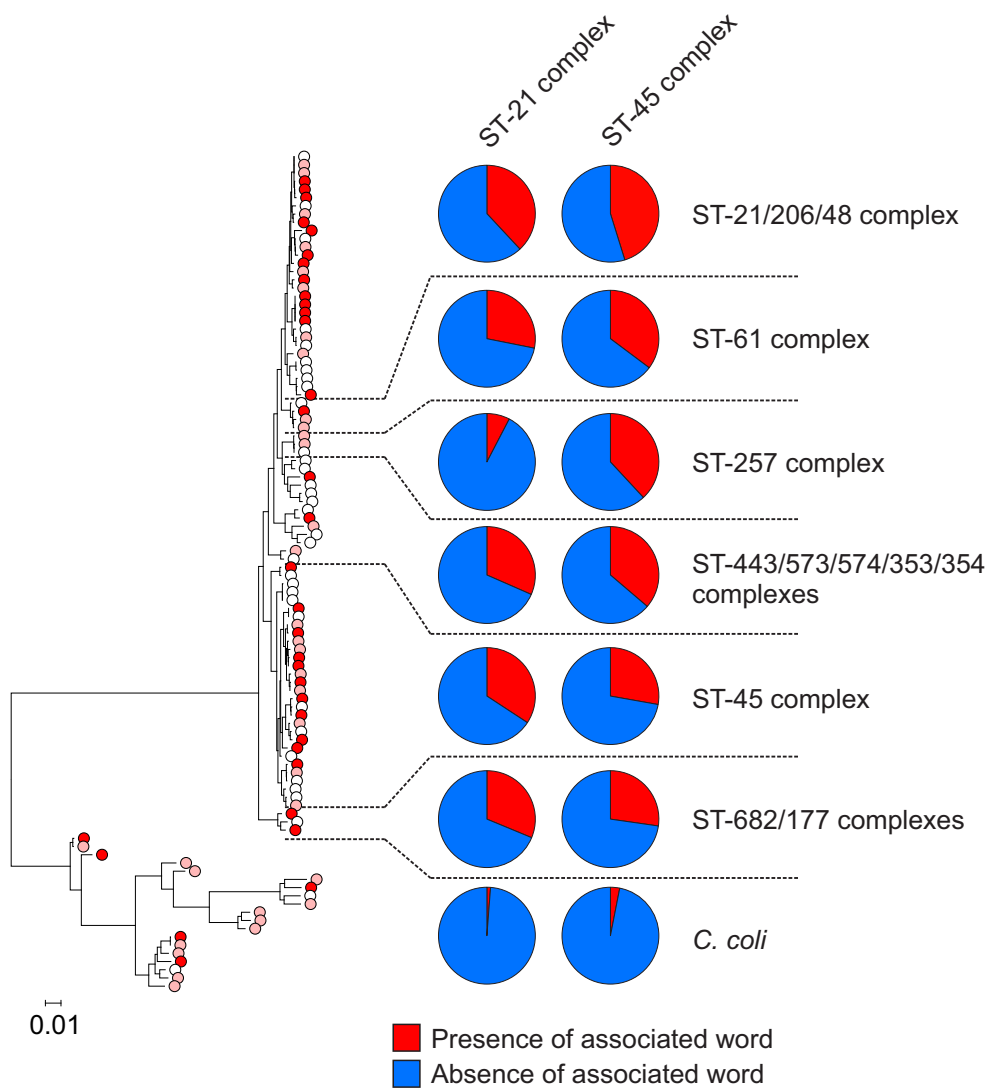

Supplement: Supplementary file 1 — Fig. S1. Distribution of biofilm absorbance readings grouped into upper (OD600 above 0.272), middle (OD600 between 0.201 and 0.272) or lower (OD600 below 0.201) 33rd percentiles. Red box plots indicate interquartile ranges. Fig. S2. The null distributions of the association scores are shown for (A) ST‐21 and (B) ST‐45 clonal complexes. In ST‐21 clonal complex, a strong population structure is indicated by a bimodal distribution, with the most frequent association scores around −7 or 7. In ST‐45 clonal complex, a normal distribution indicates weak population structure. The dashed red line indicates cut‐off corresponding to P < 0.001 in each clonal complex. Distribution of P‐values for all observed words in (C) ST‐21 and (D) ST‐45 clonal complexes. Words are not uniformly distributed because many words tend to show the same P‐values. Fig. S3. Growth of Campylobacter isolates during biofilm production under different O2 concentrations as measured by the change in absorbance (OD600). Dotted lines indicate standard errors. Growth under atmospheric (20%), 10% and 5% oxygen conditions are represented by black, red and blue lines respectively. Fig. S4. The distribution of biofilm‐associated words identified by genome‐wide association studies in other clonal complexes. The proportion of ST‐21 and ST‐45 specific biofilm‐associated words is shown as a pie chart (red indicates the presence of the associated word, blue indicates absence of the associated word) alongside a neighbour joining tree of all isolates used in the study. Isolates on the tree are coloured by their ability to form biofilm: red for an OD600 above 0.272, pink for an OD600 between 0.201 and 0.272 and white for an OD600 below 0.201. Fig. S5. Growth of Campylobacter isolates during biofilm production grouped by ecological groups as measured by the change in absorbance (OD600). Dotted lines indicate standard errors. Growth of host generalist (black), chicken specialist (orange), cattle specialist (blue) and C. c [file EMI-17-4779-s001.zip › Figure S4.pdf]

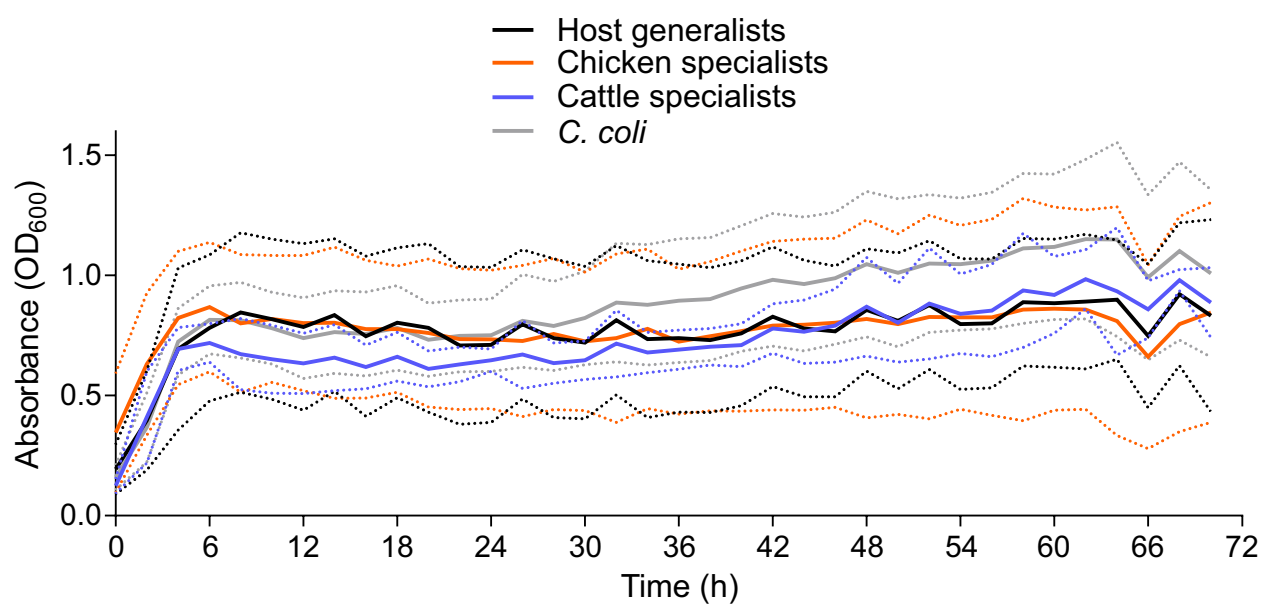

Supplement: Supplementary file 1 — Fig. S1. Distribution of biofilm absorbance readings grouped into upper (OD600 above 0.272), middle (OD600 between 0.201 and 0.272) or lower (OD600 below 0.201) 33rd percentiles. Red box plots indicate interquartile ranges. Fig. S2. The null distributions of the association scores are shown for (A) ST‐21 and (B) ST‐45 clonal complexes. In ST‐21 clonal complex, a strong population structure is indicated by a bimodal distribution, with the most frequent association scores around −7 or 7. In ST‐45 clonal complex, a normal distribution indicates weak population structure. The dashed red line indicates cut‐off corresponding to P < 0.001 in each clonal complex. Distribution of P‐values for all observed words in (C) ST‐21 and (D) ST‐45 clonal complexes. Words are not uniformly distributed because many words tend to show the same P‐values. Fig. S3. Growth of Campylobacter isolates during biofilm production under different O2 concentrations as measured by the change in absorbance (OD600). Dotted lines indicate standard errors. Growth under atmospheric (20%), 10% and 5% oxygen conditions are represented by black, red and blue lines respectively. Fig. S4. The distribution of biofilm‐associated words identified by genome‐wide association studies in other clonal complexes. The proportion of ST‐21 and ST‐45 specific biofilm‐associated words is shown as a pie chart (red indicates the presence of the associated word, blue indicates absence of the associated word) alongside a neighbour joining tree of all isolates used in the study. Isolates on the tree are coloured by their ability to form biofilm: red for an OD600 above 0.272, pink for an OD600 between 0.201 and 0.272 and white for an OD600 below 0.201. Fig. S5. Growth of Campylobacter isolates during biofilm production grouped by ecological groups as measured by the change in absorbance (OD600). Dotted lines indicate standard errors. Growth of host generalist (black), chicken specialist (orange), cattle specialist (blue) and C. c [file EMI-17-4779-s001.zip › Figure S5.pdf]
